# A prospective comparison of ER, PR, Ki67 and gene expression in paired sequential core biopsies of primary, untreated breast cancer

**DOI:** 10.1186/s12885-016-2788-x

**Published:** 2016-09-22

**Authors:** Sirwan M. Hadad, Lee B. Jordan, Pankaj G. Roy, Colin A. Purdie, Takayuki Iwamoto, Lajos Pusztai, Stacy L. Moulder-Thompson, Alastair M. Thompson

**Affiliations:** 1 St. Bartholomew’s Hospital, Barts Health, London, UK; 2 Department of Pathology, Ninewells Hospital and Medical School, Dundee, DD1 9SY UK; 3 Breast Unit, Churchill Hospital, Oxford, UK; 4 Department of Breast and Endocrine Surgery, Okayama University, Okayama, Japan; 5 Yale Medical Oncology, PO Box 208028, New Haven, 06520 CT USA; 6 Department of Breast Medical Oncology, University of Texas MD Anderson Cancer Center, 1400 Holcombe Boulevard, Houston, 77030 TX USA; 7 Department of Breast Surgical Oncology, University of Texas MD Anderson Cancer Center, 1400 Holcombe Boulevard, Houston, 77030 TX USA

**Keywords:** Breast cancer, Biomarkers, Expression arrays

## Abstract

**Background:**

Sequential biopsy of breast cancer is used to assess biomarker effects and drug efficacy. The preoperative “window of opportunity” setting is advantageous to test biomarker changes in response to therapeutic agents in previously untreated primary cancers. This study tested the consistency over time of paired, sequential biomarker measurements on primary, operable breast cancer in the absence of drug therapy.

**Methods:**

Immunohistochemistry was performed for ER, PR and Ki67 on paired preoperative/operative tumor samples taken from untreated patients within 2 weeks of each other. Microarray analysis on mRNA extracted from formalin fixed paraffin embedded cores was performed using Affymetrix based arrays on paired core biopsies analysed using Ingenuity Pathway Analysis (IPA) and Gene Set Analysis (GSA).

**Results:**

In 41 *core/resection* pairs, the recognised trend to lower ER, PR and Ki67 score on resected material was confirmed. Concordance for ER, PR and Ki67 without changing biomarker status (e.g. ER+ to ER-) was 90, 74 and 80 % respectively. However, in 23 paired *core* samples (diagnostic core v on table core), Ki67 using a cut off of 13.25 % was concordant in 22/23 (96 %) and differences in ER and PR immunohistochemistry by Allred or Quickscore between the pairs did not impact hormone receptor status. IPA and GSA demonstrated substantial gene expression changes between paired cores at the mRNA level, including reduced expression of ER pathway analysis on the second core, despite the absence of drug intervention.

**Conclusions:**

Sequential core biopsies of primary breast cancer (but not core versus resection) was consistent and is appropriate to assess the effects of drug therapy in vivo on ER, PR and Ki67 using immunohistochemistry. Conversely, studies utilising mRNA expression may require non-treatment controls to distinguish therapeutic from biopsy differences.

## Background

Biomarker studies based on the use of core biopsy and/or resection specimens for translational research in breast cancer are useful to evaluate effects of therapeutic intervention in neoadjuvant, pre-surgical and metastatic studies. Previous studies have sought differences in ER, PR and HER2 between core biopsies and resected surgical specimens in primary breast cancer and noted discordance (usually a reduction in expression) ranging from 1.2 to 35 % [1–4]. Concerns remain that core biopsy and surgical specimens may be a source of bias in clinical trials [5]. The reporting of diagnostic specimens [6] and recommendations for tumor marker prognostic studies [7] are well established with recommendations in breast cancer as to the appropriate use of tumor markers [8]. Recently, Ki67 has come to prominence as a biomarker in breast cancer of prognostic and predictive potential [9, 10].

In the clinical setting, sequential tumor core biopsy has become accepted in neoadjuvant and window of opportunity studies to seek early evidence of therapeutic efficacy [11–13]. This has included neoadjuvant endocrine trials [14, 15] and novel agents [13] or repurposing drugs [12, 16] in window of opportunity studies. The relative simplicity, accessibility and specificity of immunohistochemistry on formalin fixed, paraffin embedded (FFPE) remains attractive. Trials have identified Ki67 at 2 weeks as a predictor of relapse free survival [14] or efficacy respectively [17] and as a prognostic marker for adjuvant chemotherapy [18, 19]. Other studies have demonstrated changes in gene expression associated with response to neoadjuvant therapy [20] although signatures of response to chemotherapy have to date been rare [21].

Based on the suggestion that Ki67 may have prognostic and predictive value, the neoadjuvant Alliance ALTERNATE trial (NCT01953588) utilises changes in Ki67 after 1 month of endocrine therapy as a decision tool for subsequent continuation of endocrine therapy or switch to chemotherapy in postmenopausal women with ER positive primary breast cancer. The POETIC (Peri-operative Endocrine Treatment for Individualising Care) Trial (CR-UK/07/015) will evaluate the importance of Ki67 (and other biomarkers) after 2 weeks of treatment with a non-steroidal aromatase inhibitor in predicting long-term outcome. These, and other, clinical trials are predicated on breast cancer biopsy material reflecting therapeutic effect. However, the consistency of markers examined by immunohistochemistry [22] and (for premenopausal women) the effect of differences in the endocrine environment [23] could modify immunohistochemical and gene expression data (in the absence of therapeutic intervention) and hence may influence interpretation of drug efficacy in such settings.

Core biopsy is now considered the tumor sample of choice for ER, PR and HER2 assessment, given the excellent fixation possible [24]. The effects of tissue handling on RNA yield and integrity [25] or comparison between proteins expressed at the centre or periphery of breast cancer [26] are established. However, comparative studies for ER, PR, Ki67 or mRNA expression on paired core biopsies in the absence of therapeutic intervention are needed to test for the consistency between sequential core biopsies and to consider the potential for a wounding effect which might interfere with therapeutic assessment. This study examined paired primary breast cancer biopsies with a 2 week interval between sampling, using immunohistochemistry for ER, PR and Ki67 and mRNA gene expression.

## Methods

### Immunohistochemistry comparison between core biopsy and resection specimens

**Fig. 1:**
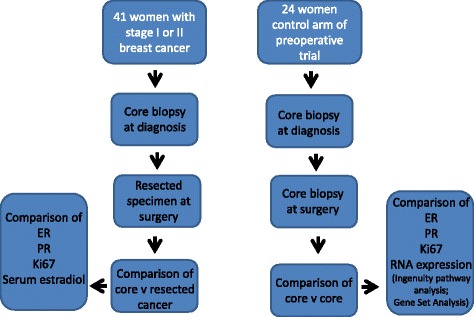
Remark diagram of patients and samples

To re-evaluate the consistency of staining between core biopsy and breast cancer resection specimens, 41 Caucasian women with histologically proven stage I or II primary breast cancer gave written, informed consent to participation under the auspices of the Tayside Local Research Ethics Committee (Fig. 1). Patients taking hormone replacement therapy (HRT) or oral contraception were excluded; 26 women were postmenopausal and 15 women premenopausal. FFPE paired biopsies at the time of diagnosis (core biopsy) and 2 weeks later at resection (from the surgical resected specimen taken at pathology cut up) were examined. The resected tumor was delivered fresh to the pathology laboratory (in under 30 min), the margins inked, the specimen sliced at 5–10 mm intervals and fixed overnight in neutral buffered formalin prior to final dissection and block selection. Core biopsies taken at the time of diagnosis were compared with tissue microarrays (TMA) made from the resected specimen. For the TMA, 6 × 0.6 mm cores of invasive disease were selected to avoid prior biopsy sites by a specialist breast pathologist. No therapeutic intervention occurred between the two sampling time points.

Immunohistochemistry was performed on 4 μm sections of FFPE tissues using standard methodologies [27] using primary antibodies for estrogen receptor alpha (ER) antibody 6 F11 (1:200; Novocastra Laboratories Ltd), progesterone receptor (PR) antibody clone 16 (1:800; Novocastra Laboratories Ltd) and NCL-L-Ki67-MM1 (Anti-Ki67, monoclonal antibody, Leica Microsystems). Negative controls (lacking primary antibody) were performed for all staining runs.

Samples were scored independently to agreement by two authors (PGR and LBJ) for an average of the cores scored- usually all six on the TMA- using the Quickscore method assessing intensity and proportion (hence for example 6 × 2 reflects % cells staining x intensity) for ER, PR [28] and using a cut off of 20 % for Ki67 [9].

### Immunohistochemistry comparison between paired core biopsies

To eliminate potential tissue handling, fixation and processing differences, core biopsies were taken 2 weeks apart (*n* = 24) from consenting patients under a separate Tayside Local Research Ethics Committee permission as control tissues from a pre-surgical metformin trial [12]. All tissues were placed immediately in neutral buffered formalin and following overnight fixation processed to paraffin blocks at a single laboratory.

For the paired cores, immunohistochemistry for ER and PR was performed as described above and scored using the Quickscore method [28] and independently by the Allred method [29]. Immunohistochemistry was conducted blinded to the clinical data and scored by a single specialist breast pathologist (LBJ). Following light microscopy review, slides were scanned into a virtual microscopy format using an Aperio ScanScope XT TM (Aperio Technologies, Vista, Ca., USA) at the x40 objective utilizing standard compression methodology.

The Ki67 index (percentage of nuclear positive cells) per invasive tumor was calculated using manual annotation of the virtual microscopy slide by means of a Wacom Bamboo Pen & Touch tablet device (Wacom Corporation, Saitama Japan) within the WebScope environment (version 10.2.0.2319) of the Aperio Spectrum Plus system version 10.2.2.2317. The annotations were assessed by the Aperio IHC nuclear Algorithm version 10. Only invasive tumor cells were assessed; great care was taken to exclude normal epithelial, in situ epithelial, stromal and inflammatory elements. A mean 5600 nuclei (range 601–39,788) per invasive tumor was assessed to obtain the Ki67 index. A minimum of 1000 invasive tumor cells was examined except for one pre-treatment and one post-treatment core (601 and 825 cells respectively).

### RNA Microarray

For RNA microarray analysis, FFPE core biopsy samples from 12 otherwise unselected patients from the control arm of a preoperative clinical trial [12] were examined. These represent 12 pairs of the 24 paired samples from the immunohistochemistry comparison between paired core biopsies where there was sufficient tumour material in the core for RNA extraction and analysisconfirmed on a Haematoxylin and Eosin slide was confirmed by a specialist breast pathologist (LBJ). RNA extraction and Breast Cancer Disease-Specific Array (DSA) gene expression profiling was performed as previously described [12].

Data were corrected for background noise, summarized and normalized using RMA in Partek® Genomics Suite™ software, 6.5 beta © 2009 (Partek Inc., St. Louis, MO, USA). Principle component analysis (PCA) revealed that the main variance associated with the first principle component was array quality. An additional transformation based in singular value decomposition was performed to remove this technical variation. The data was subsequently log2 transformed.

#### Differential gene selection

Reliably detected genes were selected by removing the probe sets with a variance below the mean global variance. The genes were then filtered based on fold change (>1.3 for less stringent and 1.5 for stringent selection) to select the differentially expressed probe sets between the second biopsy and the baseline biopsy. A student’s *t*-test without multiple testing corrections was performed and significant genes (*p*-value < 0.05 for less stringent and *p*-value < 0.005 for stringent selection) selected for further analysis.

#### Ingenuity Pathway Analysis (IPA)

Ingenuity Pathway Analysis (IPA) analysis mapped genes differentially expressed between baseline and follow-up biopsies to biological pathways using the standard commercial software (IPA, http://www.ingenuity.com)

#### Gene Set Analysis (GSA)

Gene Set Analysis (GSA) examined whether members of a particular biological pathway occur toward the top or the bottom of a rank-ordered gene list including all gene expression measurements ranked by differential expression between baseline and second core biopsy. This analysis takes into account information from members of a pathway that would not make it to the top most differentially expressed gene list (used for the IPA analysis above). GSA was performed using the BRB Array Tools software package (http://linus.nci.nih.gov/BRB-ArrayTools.html, US NCI Biometrics Branch) for 2987 gene sets collectively representing most known biological and metabolic pathways in Gene Ontology (GO, http://www.geneontology.org). To be included, a GO gene set required a minimum of 10 and a maximum of 200 genes. Significance was estimated with a permutation test (*n* = 1000). The null hypothesis was that the average degree of differential expression of members of a given gene set between the baseline and second biopsy was the same as expected from a random permutation of biopsy labels. IPA software was used to generate pathway figures for the significant gene sets.

## Results

### Comparison between core biopsy and resection specimens

**Fig. 2:**
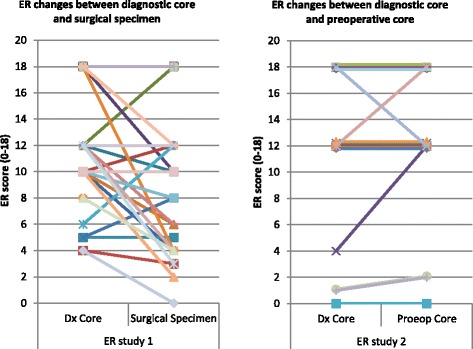
Estrogen receptor expression by IHC on sequential specimens (core v resection, *left panel*, core v core, *right panel*)

**Fig. 3:**
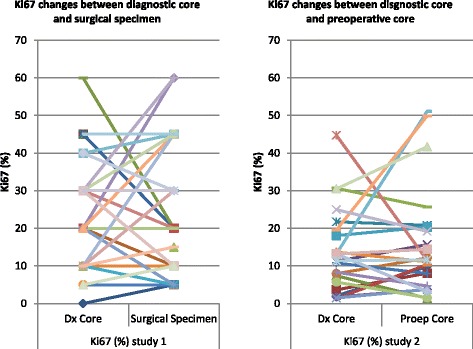
Ki67 expression by IHC on sequential specimens (core v resection, *left panel*, core v core, *right panel*)

In tumor samples from 41 women (Table 1) there was a clinically significant change (loss) of ER between the diagnostic core and the resection specimen in cancers from 4/41 (10 %) women across the threshold for adjuvant endocrine therapy of a Quickscore of 4/18, although the ER score changed in a further 18 women, but would not change the clinical impact (Fig. 2 and Table 2). Loss of ER was identified in 3/15 (20 %) premenopausal women and PR changes occurred in both premenopausal and postmenopausal women. For Ki67 (Fig. 3), there was also a loss of staining in assessable samples to below 20 % in 1/15 (7 %) premenopausal and 4/25 (16 %) postmenopausal women and a rise above 20 % in 2/15 (14 %) premenopausal and 1/25 (4 %) postmenopausal women; Ki67 was not assessable on one core.

**Table 1:** Changes in ER, PR and Ki67 in paired core biopsy/resection specimens (*n* = 42 women)

	Number of patients	Change from <4 to ≥4	Change from ≥4 to < 4	Rise of fall in score, but not crossing threshold 4	No change between samples
ER premenopausal	15	0	3	4	8
ER postmenopausal	26	0	1	14	11
PR premenopausal^a^	15	1	4	4	6
PR postmenopausal^a^	16	1	3	6	6
		Change from <20 % to ≥20 %	Change from ≥20 % to <20 %	Rise or fall in Ki67, but not crossing 20 %	No change between samples
Ki67 premenopausal	15	2	1	8 rise	4
Ki67 postmenopausal^b^	25	1	4	3 rise + 11 fall	6

Notes

^a^PR not assessed in the diagnostic core from one premenopausal and nine postmenopausal women

^b^Ki67 not assessed in the diagnostic core from two postmenopausal patients

**Table 2:** Comparison of ER and PR in paired core biopsies (*n* = 23 women)

	No change	Reduced expression (no switch)^a^	Increased expression (no switch)^a^	Switch^b^	Missing data
ER	17	2	3	0	6
PR	17	6	3	0	6

Notes

^a^No switch either by Allred score or Quickscore

^b^Switch only using Allred score

### Immunohistochemistry comparison between paired core biopsies

In paired core biopsies from 17 women, using the Quickscore method, in 2/17 (12 %) there was reduced expression of ER in the second core biopsy and in 3/17 (18 %) increased expression of ER in the second core (Fig. 2). In none of these five patients would the change in ER have led to a therapeutically important switch whether the Quickscore or Allred score was applied.

For PR in 6/17 (35 %) women there was reduced expression of PR in the second core biopsy and in 3/17 (18 %) increased expression of PR in the second core. In none of these nine patients would the change in PR have led to a therapeutically important switch whether the Quickscore or Allred score was used.

Ki67 was available on 23 paired core biopsies (including the 17 for ER and PR pairs). Using 20 % as a cut off [9], 5/23 (22 %) tumor samples would have crossed the 20 % threshold between the paired samples: 2/23 (9 %) patients would have crossed from above to below 20 % and tumor samples from a further 3/23 (13 %) patients from below to above 20 %. However, using 13.25 % as the cut off [10], only 1/23 (4 %) tumors would have crossed the 13.25 % boundary comparing the two cores (Fig. 3).

### RNA microarray

Microarray analysis was successfully completed on all 12 paired samples. By paired *t*-test differences in gene expression profile were identified between the diagnostic and surgical core biopsy.

**Fig. 4:**
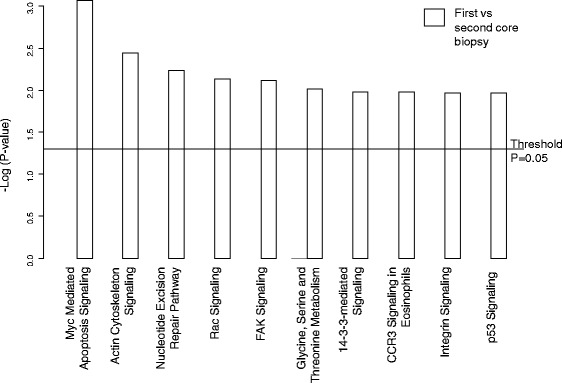
Cell pathways associated altered between sequential core biopsies

**Fig. 5:**
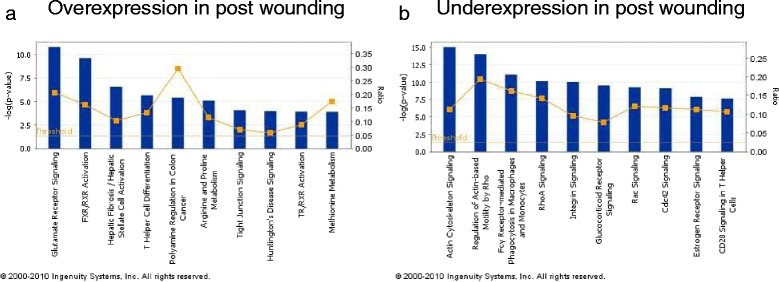
Cellular pathways associated with wounding effect by GSA. Cell pathways (**a**) overexpressed between sequential core biopsies and (**b**) underexpressed between sequential core biopsies

By GSA (Fig. 4), the differences between the two biopsies suggested changes in pathways involving myc, apoptosis and p53 amongst others in the second biopsy compared with the first. Several elements of cellular metabolism and immunological pathways were identified as overexpressed (Fig. 5a) in the second biopsy as compared with the first whereas, the Rho, integrin and potentially significantly the ER pathways were relatively underexpressed (Fig. 5b) in the second core biopsy.

IPA set in context a number of gene expression changes among which pathways involving PI3K, MEKK and IGF-1 may be of particular relevance in the setting of breast cancer.

## Discussion

Minimising bias in clinical molecular marker studies in preoperative trials using paired samples is critical to assess the efficacy and target effects of endocrine agents (for example the ALTERNATE and POETIC trials), novel therapy [13] or new indications for established drugs [12] and to change clinical management, at least in the trial setting (ALTERNATE).

### Immunohistochemistry comparison between core biopsy and resection specimens

**Fig. 6:**
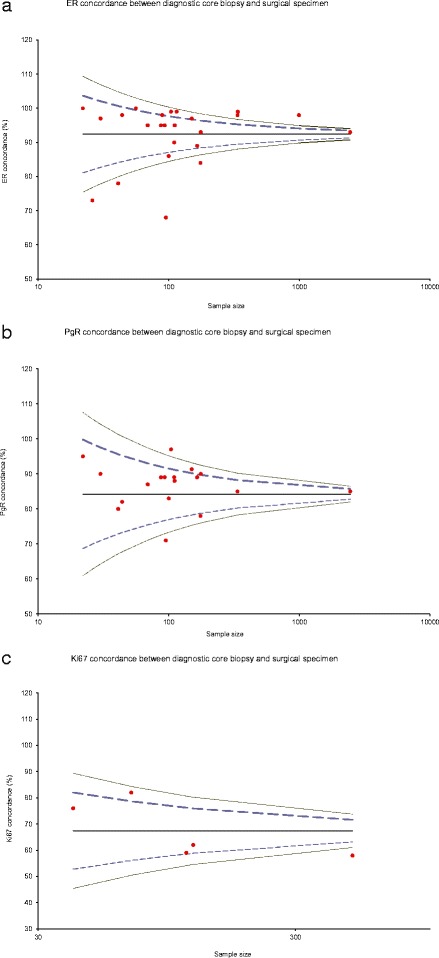
**a** Funnel plot for 24 studies on ER concordance between diagnostic cores and surgical specimen. Mean concordance is 92.38 %. Excluding the seven studies that fall outside the 99 % Confidence Interval, changed the mean to 95.63 %. **b** Funnel plot for 19 studies on PgR concordance between diagnostic cores and surgical specimen. Mean concordance is 84 %. Excluding the two studies that fall outside the 99 % Confidence Interval has not changed the mean. **c** Funnel plot for five studies on Ki67 concordance between diagnostic cores and surgical specimen. Mean concordance is 67.4 %. Excluding the study that fall outside the 99 % Confidence Interval, changed the mean to 69.75 %

To date there have been multiple comparisons of core biopsies and surgical resections for ER, PR, Ki67 for tumor grade and HER2 (Table 3) demonstrating a mean concordance of 92.4 % for ER (Fig. 6a), 84 % for PR (Fig. 6b) and 67.4 % for Ki67 (Fig. 6c), comparable to the data presented here. Reporting comparisons between ER, Ki67 and other biomarkers in this setting may be potentially misleading for well-rehearsed reasons [1, 5, 30] minimised by the use of (paired) core biopsies and consistent tissue handling. We revisited whether the changes in ER might be secondary to changes in circulating estradiol, confirming plausible evidence for premenopausal women [23], but likely due to tissue handling and processing at least in postmenopausal women [1, 5, 25].

**Table 3:** Published research articles on concordance between diagnostic core biopsies and surgical specimens for tumour grade, Ki67, ER, PgR and Her2

Authors	Sample size	Tumour Grade (%)	Ki67 (%)	Tumour type (%)	ER (%)	PgR (%)	HER2 (%)
Motamedolshariati et al. (2014) [36]	30	67		100	97	90	93
Munch-Peterson et al. (2014) [37]	89	77			98		84
Loubeyre et al. (2013) [38]	993				98		
Dekker et al. (2012) [39]	115				99		96.2
Greer et al. (2012) [40]	165				89	89	93
Lee et al. (2012) [41]	300						98
Li et al. (2012) – meta-analysis [4]	2450				93	85	
Ricci et al. (2012) [42]	69		82		95	87	78
Khoury et al. (2011) [43]	176				93	90	
Lorgis et al. (2011) [44]	175	75			84	78	98
Arnedos et al. (2009) [3]	336				98	85	99
Park et al. (2009) [45]	104	81		100	99	97	86
Usami et al. (2007) [46]	111	75		83	95	88	88
Cahill et al. (2006) [47]	95	77		98	68	71	60
Burge et al. (2006) [48]	87	77		100	95	89	96
Hodi et al. (2007) [49]	338				99		
Badoual et al. (2005) [50]	110	73.1		74	90	89	
Usami et al. (2005) [51]	22	80		89	100	95	80
Al Sarakbi et al. (2005) [52]	93				95	89	
Mann et al. (2005) [1]	100				86	83	80
Deshpande et al. (2005) [53]	105	75		96			
O'Leary et al. (2004) [54]	113	62	59	65			
Andrade and Gobbi (2004) [55]	120	59	62	67			
Harris et al. (2003) [56]	500	67	58	74			
Connor et al. (2002) [57]	44	64			98	82	91
McIntosh et al. (2002) [58]	133	91		84			
Sharifi et al. (1999) [59]	79	75		81			
Gotzinger et al. (1998) [60]	150	84		100	97	91.3	
Jacobs et al. (1998) [61]	56				100		100
Di Loreto et al. (1996) [62]	41	80	76		78	80	90
Dahlstrom et al. (1996) [63]	51	69		78			
Baildam et al. (1989) [64]	140	69					
Zidan et al. (1997) [65]	26				73	42

### Immunohistochemistry comparison between paired core biopsies

Paired core biopsies of primary breast cancer before/after drug therapy has become popular [12, 13, 16], although quality standards for Ki67 have been of concern [9, 10]. In a trial setting [12], variations in specimen processing, specimen handling, laboratory processing and immunohistochemical staining and scoring were minimised, although patient selection (ER positive T1c and T2 cancers) occurred.

Slight variation of immunohistochemical scoring of ER and PR between paired cores, potentially attributable to geographic targeting differences over time, rarely crossed the boundary for clinical decision making. For Ki67, the cut point was key: at 20 % [9], 5/23 (22 %) paired tumor samples would have crossed the threshold, compared with only 1/23 (4 %) tumors using 13.25 %, in concordance with expert opinion [10] confirming a Ki67 boundary of 13.25 % is appropriate when seeking evidence of a drug effect.

While intra-tumoral heterogeneity has been considered elsewhere [26], the single cores at each time point may reflect clinical reality in small cancers for window of opportunity, pre-operative or neoadjuvant trials. Given the consensus, for a number of tumor types, that needle biopsy specimens result in reliable immunohistochemistry [1, 31], this study provides reassurance that immunohistochemical measurement of ER, PR and Ki67 from core biopsy pairs is consistent over 2 weeks.

### RNA microarray

By GSA, the changes expression of genes integral to cell cycle and apoptosis (Fig. 4), overexpression of cellular metabolism and immunological pathways (Fig. 5a) and underexpression of cell motility and cell adhesion (Fig. 5b) suggest that in the time frames of the biopsy, perturbation of such pathways remains several days after the initial wounding effect of the first core biopsy. The reduction in mRNA expression of the ER pathway (Fig. 5b) following the first biopsy holds potential concern and is in contrast to the only other published study of eight patients where no change was noted [32]. However, mRNA changes do not exactly reflect semiquantiative immunohistochemistry and ER mRNA imperfectly correlates with the level of ER protein expression [33]. The immunohistochemical studies on the same series of samples reported here provide comfort that for the technology most widely used in clinical practice (immunohistochemistry), ER on a second core biopsy may not be compromised.

IPA set in context a number of gene expression changes among which pathways involving PI3K, MEKK and IGF-1 [34, 35] may be of particular relevance in the setting of breast cancer.

These microarray data, within the limits of the experimental design, sample numbers and analytical techniques employed, suggest that core biopsy of primary breast cancer may generate a “wounding” effect evident on subsequent mRNA analysis. The time course, duration and variations in gene expression as a consequence of tumor and patient variability were not assessed within this study and are clinically challenging to obtain [25]. However, core biopsy may influence the mRNA expression profile of sequential clinical samples used in clinical trials and requires careful evaluation.

## Conclusions

This study provides reassurance that sequential core biopsy (but not core versus resection) should be an appropriate way to assess the effects of drugs on primary tumor ER, PR and Ki67 (with a cut off of 13.25 %) within the context of window of opportunity and neoadjuvant trials. By contrast, mRNA analyses may demonstrate multiple changes between paired samples reflecting the wounding effect of core biopsy, which for ER at least is not reflected at the level of immunohistochemistry. Sequential core biopsy may be used with confidence when seeking evidence of ER, PR and Ki67 changes in the preoperative setting for primary breast cancer.

## Abbreviations

DSA: Disease Specific Array
ECLIA: Electrochemoluminescence immunoassay
ER: Estrogen receptor
FFPE: Formalin fixed paraffin embedded tissue
GSA: Gene Set Analysis
HER2: Human epidermal growth factor type 2
HRt: Hormone replacement therapy
IGF-1: Insulin like growth factor-1
IPA: Ingenuity Pathway Analysis
MEKK: Mitogen activated protein kinase kinase
mRNA: Messenger RNA
PCA: Principle component analysis
PI3K: Phosphoinositol-3-kinase
PR: Progesterone receptor
TMA: Tissue MicroArray
